# OsHrd3 is necessary for maintaining the quality of endoplasmic reticulum-derived protein bodies in rice endosperm

**DOI:** 10.1093/jxb/erv229

**Published:** 2015-05-14

**Authors:** Masaru Ohta, Fumio Takaiwa

**Affiliations:** Functional Transgenic Crops Research Unit, Genetically Modified Organism Research Center, National Institute of Agrobiological Sciences, Kannondai 2-1-2, Tsukuba, Ibaraki 305–8602, Japan

**Keywords:** Prolamins, protein body, protein quality control, seed storage protein, unfolded protein response.

## Abstract

Large amounts of seed storage proteins (SSPs) are produced in the maturing endosperm of rice seeds. Rice SSPs are synthesized as secretory proteins on the rough endoplasmic reticulum (ER), and are transported and deposited into protein complexes called protein bodies (PB-I and PB-II). Due to the high production of SSPs, unfolded SSPs may be generated during this process. However, it was previously unclear how such unfolded proteins are selected among synthesized products and removed from the ER to maintain protein quality in the endosperm. Since Hrd3/SEL1L recognizes unfolded proteins in yeast and mammalian protein quality control systems, the role of OsHrd3 in protein quality control in rice endosperm was investigated. Co-immunoprecipitation experiments demonstrated that OsHrd3 interacts with components of the Hrd1 ubiquitin ligase complex such as OsOS-9 and OsHrd1 in rice protoplasts. Endosperm-specific suppression of *OsHrd3* in transgenic rice reduced the levels of polyubiquitinated proteins and resulted in unfolded protein responses (UPRs) in the endosperm, suggesting that OsHrd3-mediated polyubiquitination plays an important role in ER quality control. It was found that a cysteine-rich 13kDa prolamin, RM1, was polyubiquitinated in wild-type (WT) seeds but not in *OsHrd3* knockdown (KD) seeds. RM1 formed aberrant aggregates that were deposited abnormally in *OsHrd3* KD seeds, resulting in deformed PB-I. Therefore, the quality of protein bodies is maintained by polyubiquitination of unfolded SSPs through the Hrd1 ubiquitin ligase system in rice endosperm.

## Highlight

OsHrd3 plays crucial roles in protein quality control of seed storage proteins through polyubiquitination of a cyteine-rich prolamin, RM1, during rice seed maturation.

## Introduction

Seed storage proteins (SSPs) are a source of the nitrogen, sulphur, and carbon required for the germination and growth of seedlings prior to photosynthesis. Rice SSPs are synthesized on the rough endoplasmic reticulum (rER) and translocated into the ER lumen, followed by deposition in two different types of protein bodies, PB-I and PB-II (Tanaka *et al.*, 1980; Krishnan *et al.*, 1986). PB-I is a 1–2 μm, spherical proteinaceous granule derived from the ER. PB-I is composed of cysteine-rich (10, 13, and 16kDa) and cysteine-poor (13kDa) prolamins (Ogawa *et al.*, 1987). PB-II is an irregularly shaped, 2–4 μm protein storage vacuole that has high electron density. PB-II is formed via the Golgi apparatus or by precursor-accumulating (PAC) vesicles from the ER, into which glutelins and globulin are deposited (Takahashi *et al.*, 2005). Glutelin is synthesized as proglutelin in the ER lumen and subsequently processed into mature acidic and basic subunits in PB-II. Large amounts of SSPs are produced in the ER of rice endosperm and, thus, the ER in rice endosperm is densely packed with polypeptides. In such a crowded cellular environment, unfolded proteins are often produced through stochastic errors during protein synthesis or by perturbation due to adverse environmental changes (Gershenson and Gierasch, 2011; Hartl *et al.*, 2011). The accumulated unfolded proteins may form aggregations, resulting in perturbed cellular homeostasis when these unfolded proteins bind to other proteins. However, there may be unknown molecular mechanisms that serve to clean up the unfolded proteins and to maintain functional, healthy proteostasis in the ER of rice endosperm.

Protein quality control systems are mechanisms used to maintain healthy proteostasis in the ER (Brodsky, 2012). ER chaperones, folding enzymes, and chaperone-like proteins play central roles in protein quality control, and these proteins mediate the repair of improperly/incompletely folded polypeptides. In contrast, severely damaged unfolded polypeptides are recognized and sequestered for degradation via a process known as ER-associated degradation (ERAD). The unfolded polypeptides are then retrotranslocated from the ER to the cytoplasm and marked with ubiquitins by a catalytic cascade of E1 (ubiquitin-activating enzyme), E2 (ubiquitin-conjugating enzyme), and E3 (ubiquitin ligase) activities (Hochstrasser, 1996; Hershko and Ciechanover, 1998). Since proteosome delivery and degradation require the ubiquitination of the substrate, unfolded polypeptides, the ubiquitination enzymes are crucial for ERAD. Finally, polyubiquitinated, unfolded polypeptides are degraded by proteosomes.

Increasing numbers of components of the ERAD machinery have been identified in plants. The AAA ATPase Cdc48/p97 is a direct contributor to the retrograde translocation of ERAD substrates at an intermediate step preceding proteosomal protein degradation in mammalian cells and yeast (Braun *et al.*, 2002; Jarosch *et al.*, 2002). Plant Cdc48 is involved in the retrograde transport of plant ERAD substrates such as the mutant forms of mildew resistance o (MLO) protein and mutated ricin (Müller *et al.*, 2005; Marshall *et al.*, 2008). The isolation of suppressor mutants for a brassinosteroid-insensitive mutant revealed that a number of ERAD-related genes similar to those in yeast and mammals are involved in the degradation of the mutated brassinosteroid receptor. These include genes encoding UDP-glucose:glycoprotein glucotransferase (UGGT), a plant-specific calreticulin, and a homologue of yeast Hrd3/mammalian SEL1L (Jin *et al.*, 2007, 2008; Su *et al.*, 2011). Hrd3/SEL1L is a component of the Hrd1 E3 ligase complex and is involved in substrate recruitment (Carvalho *et al.*, 2006; Denic *et al.*, 2006). *Arabidopsis* Hrd3 is necessary for the degradation of mutated brassinosteroid receptor BRI, which is implicated in the HRD pathway in plants (Su *et al.*, 2011). Furthermore, ER-resident chaperones such as calnexin and BiP interact with the mutated brassinosteroid receptors Bri1-5 and Bri1-9 (Jin *et al.*, 2007; Hong *et al.*, 2008). *Arabidopsis* ubiquitin conjugase UBC32 is an ERAD component that is involved in brassinosteroid-mediated salt stress tolerance (Cui *et al.*, 2012). These studies clearly demonstrate that plants have ERAD machinery that can degrade aberrant proteins caused by genetic mutations. However, it is still unclear whether the plant ERAD machinery is involved in protein quality control and the degradation of unfolded proteins derived from wild-type (WT) proteins, which is caused by stochastic errors during protein synthesis or perturbation by adverse environmental changes under normal conditions.

In this work, the roles of OsHrd3 in protein quality control in rice endosperm were investigated. Transgenic rice with suppressed expression of *OsHrd3* under the control of an endosperm-specific promoter were generated. The results reveal that OsHrd3 is required for the polyubiquitination of unfolded proteins, including the cysteine-rich 13kDa prolamin RM1, in rice endosperm. Thus, significant amounts of unfolded RM1 are produced under normal conditions, and the removal of unfolded RM1 is achieved through the involvement of OsHrd3, which is required for proper formation of PB-I in rice endosperm.

## Materials and methods

### Construction of pasmids

To make the following plasmid constructs, the coding region of *OsHrd3* was amplified from a rice full-length cDNA clone (AK067004) by PCR using the primers shown in Supplementary Table S1 available at *JXB* online. The Ubip-3× FLAG-GluBter vector was constructed by inserting the 3× FLAG tag fragment of the 2×35S-3× FLAG-Nos vector (Ohta *et al.*, 2013) into the *Kpn*I and *Sac*I sites of the Ubip-GFP-GluBter vector. To produce OsHrd3-FLAG, the coding region of *OsHrd3* was excised from OsHrd3–green fluorescent protein (GFP) by digestion with *Xma*I and inserted into the *Xma*I site of the Ubip-3× FLAG-GluBter vector. The 2×HA tag sequence fragment was amplified using the primers shown in Supplementary Table S1, and the DNA fragment was inserted into the *Kpn*I and *Sac*I sites of the Ubip-GluBter and the 2×35S-Nos vectors to produce the Ubip-2×HA-GluBter and the 2×35S-2×HA-Nos vectors, respectively. The coding regions of *OsHrd1* and *OsOS9* were amplified by PCR using the primers listed in Supplementary Table S1. The DNA fragments were inserted into the *Kpn*I site of the Ubip-2×HA-GluBter vector to produce OsHrd1-HA. OsOS9-HA was constructed by inserting the PCR fragment into the *Nco*I site of the 2×35S-2×HA-Nos vector.

Endosperm-specific knockdown lines for OsHrd3 (*OsHrd3* KD) were generated by RNA interference. The gene fragment containing coding sequences (base pairs 1843–2087) and 357bp of 3′ untranslated region (UTR) in *OsHrd3* was amplified by PCR using the primers listed Supplementary Table S1 at *JXB* online, and connected with the intron sequence from the rice aspartic protease gene (Kuroda *et al.*, 2010) to express intron-containing hairpin RNA. The construct was linked downstream of the 16kDa prolamin promoter and inserted into a modified pHm43GW binary vector (Wakasa *et al.*, 2011).

### Rice transformation

Transgenic rice plants (*Oryza sativa* L. cv. Kita-ake) were generated by *Agrobacterium*-mediated transformation (Goto *et al.*, 1999), and lines exhibiting suppressed expression of *OsHrd3* were screened by real-time PCR (RT-PCR) analysis of *OsHrd3* transcript levels in developing transgenic seeds [14 days after flowering (DAF)]. The T_3_ generation of homozygous plants of a representative line (line 3) was analysed.

### Immunoprecipitation experiments

The transient expression assay was carried out as described previously (Kawakatsu *et al.*, 2009). Protoplasts were prepared from cultured rice cells (Oc cells). To analyse the interaction between OsHrd3 and other proteins, co-immunoprecipitation (Co-IP) experiments were carried out essentially as described (Ohta *et al.*, 2013), except that 1% digitonin was used in the extraction buffer instead of 0.5% Triton X-100.

### Protein extraction and immunoblot analysis

Extraction of total proteins from mature WT and transgenic rice seeds and immunoblot analysis were performed as described previously (Ohta *et al.*, 2013). To analyse the aggregates, total proteins were extracted from mature WT and *OsHrd3* KD seeds using SDS–urea buffer without 2-mercaptoethanol. The extracts were centrifuged at 20 400 *g* for 10min at room temperature, and the supernatants were collected (fraction S). The pellets were again extracted with SDS–urea buffer containing 5% 2-mercaptoethanol, and the soluble fractions were collected by centrifugation as described above (fraction P). Before SDS–PAGE analysis, the proteins in the S fraction were reduced in SDS–urea buffer containing 5% 2-mercaptoethanol. Rabbit polyclonal antibodies (anti-OsBiP1, anti-OsBiP4&5, anti- OsPDIL2-3, anti-GluA, anti-GluB, anti-GluC, anti-Glb, anti-10k, anti-16k, anti-RM, anti-RM2, anti-RM4, and anti-RM9) were prepared previously (Yasuda *et al.*, 2006; Oono *et al.*, 2010; Wakasa *et al.*, 2012).

### RNA extraction and RT-PCR analysis

Total RNA was extracted from seeds as previously described (Takaiwa *et al.*, 1987). RT-PCR analysis was carried out as described (Wakasa *et al.*, 2012) using Go-Taq polymerase (Promega, WI, USA) with gene-specific primers for *OsbZIP39*, *OsbZIP50*, *OsbZIP60*, *OsBiP4*, and *17S rRNA* (Hayashi *et al.*, 2012), and for *OsHrd3* (listed in Supplementary Table S1 at *JXB* online).

### Confocal immunohistochemical analysis

Maturing WT and *OsHrd3* KD seeds were harvested at 18 DAF and used for immunocytochemical analysis as described (Ohta *et al.*, 2013).

### Detection and immunoprecipitation of polyubiquitinated proteins

Developing seeds (14 DAF) from WT and *OsHrd3* KD plants were surface-sterilized with 50% hypochlorous acid for 30min, and the hulls were aseptically removed from the sterilized seeds. The dehulled seeds (eight grains) were incubated in half-strength Murashige and Skoog (MS) liquid medium containing 100 μM MG132 overnight and then incubated in MS liquid medium containing 20 μM PR-619 b (Seiberlich *et al.*, 2012) for 1h at 28 °C with gentle shaking. Total proteins were extracted from the treated seeds with SDS–urea buffer containing 5% 2-mercaptoethanol. The extracts were subjected to immunoblot analysis using polyclonal rabbit antibodies against ubiquitin–protein conjugates (Enzo Life Science, NY, USA).

For immunoprecipitation of polyubiquitinated proteins, dehulled seeds treated with MG132 and PR-619 were homogenized in 800 μl of extraction buffer containing 50mM TRIS-HCl, pH 7.5, 150mM NaCl, 0.5% Triton X-100, 5mM EDTA, 20mM *N*-ethylmaleimide, and 1× Complete mini EDTA-free Protease Inhibitor Cocktail (Roche, Switzerland). The homogenates were centrifuged at 20 400 *g* for 10min at 4 °C and the supernatants were collected. The supernatants were mixed with immobilized anti-multiubiquitin mAb-magnetic beads (MBL, Japan) for 3h at 4 °C to immunoprecipitate the polyubiquitinated proteins. The beads were washed three times with NET buffer containing 50mM TRIS-HCl, pH 7.5, 150mM NaCl, and 0.1% NP-40. The immunoprecipitated samples were eluted with SDS loading buffer containing 2% SDS, 62.5mM TRIS-HCl pH 6.8, and 5% 2-mercaptoethanol. The samples were denatured at 65 °C for 10min and subjected to immunoblot analysis.

## Results

### Interaction between OsHrd3 and both OsHrd1 and OsOS-9

A database survey revealed that the rice genome encodes a homologue of Hrd3/SEL1L (Os03g0259300) containing a signal peptide, nine Sel1-like repeats, and a C-terminal transmembrane anchor. Thus, Os03g0259300 was designated as OsHrd3. It was confirmed that OsHrd3 is an ER-resident type I membrane protein (Supplementary Fig. S1 at *JXB* online).

**Fig. 1.**
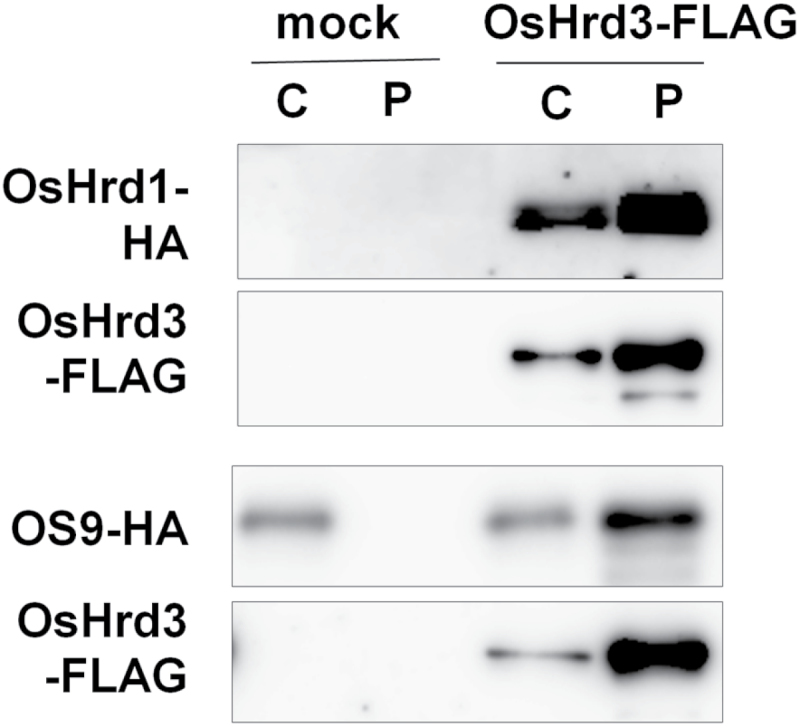
Interaction between OsHrd3 and components of the Hrd1 ubiquitin ligase complex. Immunoprecipitation of OsHrd3-FLAGs. Protein extracts from rice protoplasts expressing OsHrd1-HA, OsOS-9-HA, and OsHrd3-FLAG were subjected to immunoprecipitation using anti-FLAG M2 magnetic beads. The immunoprecipitates were analysed by immunoblot analysis using anti-FLAG–horseradish peroxidase (HRP) and anti-HA–HRP conjugated antibodies. C represents 2% (v/v) of the starting crude lysate used for immunoprecipitation. P represents immunoprecipitated proteins.

Hrd3/SEL1L is a component of the Hrd1 ubiquitin ligase complex. In yeast, the Hrd1 complex consists of Hrd3p and Der1p, including the ER lectin Yos9p bound to Hrd3p (Carvalho *et al.*, 2006; Denic *et al.*, 2006). Rice genes encoding homologues of the yeast Hrd1 and Yos9p were found in the database (RAP-DB, http://rapdb.dna.affrc.go.jp/). To examine the possibility that OsHrd3 also forms a complex with these proteins, the interaction between OsHrd3 and both OsHrd1 (Os06g0301000) and OsOS-9 (Os06g0644800), which are putative homologues of yeast Hrd1 and Yos-9p, respectively, was investigated. OsHrd1 and OsOS-9 were fused with 2× HA tag sequence at their C-termini. Plasmid DNA harboring OsHrd3-FLAG, together with OsHrd1-HA or OsOS-9-HA, was then transfected into rice protoplasts and Co-IP analysis was performed with an antibody against FLAG tag (Ohta *et al.*, 2013). As shown in Fig. 1, OsHrd1-HA and OsOS-9-HA co-precipitated with OsHrd3-FLAG, demonstrating that OsHrd1 and OsOS-9 can interact with OsHrd3. These data suggest that OsHrd3 could form a complex with OsHrd1 and OsOS-9.

### OsHrd3 is involved in polyubiquitination in rice endosperm

**Fig. 2.**
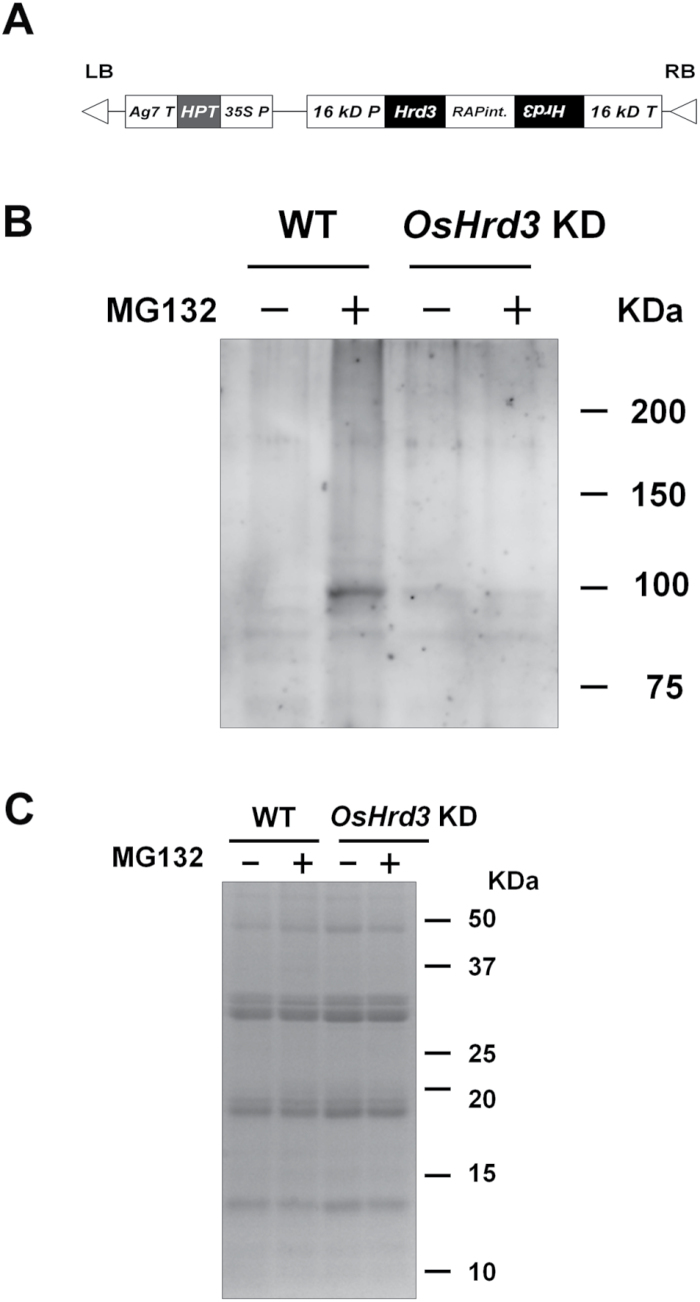
OsHrd3 is required for polyubiquitination of unfolded proteins in rice endosperm. (A) The construct used for *OsHrd3* knockdown (*OsHrd3* KD); 35S P, *Cauliflower mosaic virus* 35S promoter (AF485783); HPT, hygromycin phosphotransferase coding region (K01193); Ag7 T, gene 7 terminator (AF85783); 16 kD P, promoter region of the gene encoding 16kDa prolamin (AY427574); RAPint, an intron from the rice aspartic protease gene (D32165); 16 kD T, 16kDa prolamin terminator. (B and C) Levels of polyubiquitinated proteins are reduced in *OsHrd3* KD seeds. Seeds (14 DAF) from wild-type (WT) and *OsHrd3* KD plants were dehulled and treated with either 0.1% dimethylsulphoxide (–) or 100 μM MG132 (+) for 24h and then treated with 20 μM PR-619 for 1h. Then, total proteins were extracted from the seeds with SDS–urea buffer containing 2-mercaptoethanol. The total proteins were separated by SDS–PAGE, followed by immunoblot analyses using an antibody against ubiquitin–protein conjugates (B) or Coomassie Brilliant Blue staining (C).

**Fig. 3.**
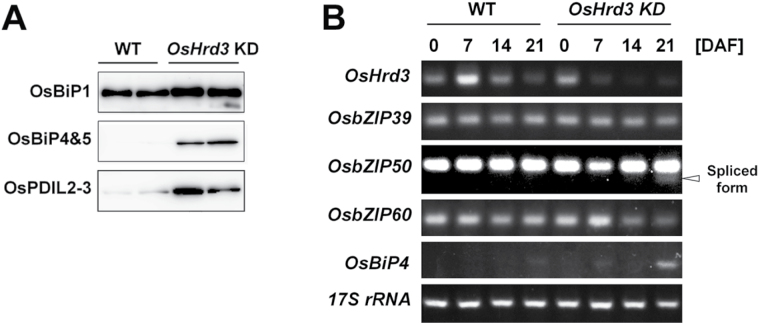
Unfolded protein responses in *OsHrd3* KD seeds. (A) Immunoblot analysis of total proteins extracted from mature WT and *OsHrd3* KD seeds. Total proteins were extracted from mature seeds with SDS–urea buffer containing 2-mercaptoethanol. The total proteins were separated by SDS–PAGE, followed by immunoblot analyses using antibodies against ER-resident chaperones. The two tracks in the WT and *OsHrd3* KD represent different samples from different seeds. (B) Induction of ER stress-related genes during seed development in WT and *OsHrd3* KD seeds. Total RNAs were isolated from seed tissues at 0, 7, 14, and 21 days after flowering (DAF). Transcript levels were estimated by semi-quantitative RT-PCR. *17S rRNA* was analysed as a loading control. The arrowhead indicates mature transcript produced by unconventional splicing of precursor *OsbZIP50* transcript.

Maturing rice seeds produce a large amount of SSPs. Thus, a significant amount of unfolded protein is likely to be produced by stochastic errors during protein synthesis, perturbation by adverse environmental changes, and an imbalance in stoichiometry among components of the protein bodies. However, it is unclear how the ER in developing seeds discriminates and removes such unfolded proteins. To elucidate the role of a quality control system in rice seeds, transgenic rice plants were generated with suppressed expression of *OsHrd3* in the endosperm under the control of the 16kDa prolamin (Os03g0766200) promoter (Fig. 2A). Since mRNAs for OsHrd3 and 16kDa prolamin were detected at 7DAF (Fig. 3B; Supplementary Fig. S2D at *JXB* online), the 16kDa prolamin promoter is suitable for suppression of OsHrd3 expression. RT-PCR analysis showed that the level of *OsHrd3* transcript was lower in *OsHrd3* KD seeds than in WT seeds (Fig. 3B).

To examine the possibility that OsHrd3 is involved in the ubiquitination of unfolded proteins in rice endosperm, the levels of polyubiquitinated proteins in rice endosperm were analysed. When maturing seeds were harvested at 14 DAF from WT and *OsHrd3* KD plants and treated with the proteosome inhibitor MG132 and the deubiquitinase inhibitor PR-619, the levels of polyubiquitinated proteins increased in the treated WT only compared with the non-treated control WT and *OsHrd3* KD seeds (Fig. 2B, C). These results demonstrate that OsHrd3 is involved in the polyubiquitination of unfolded proteins in rice endosperm, and this protein is a component of the Hrd1 ubiquitin ligase complex.

### Induction of unfolded protein response in *OsHrd3* KD seeds

Since the polyubiquitination of unfolded proteins is impaired in *OsHrd3* KD seeds, the unfolded proteins in these seeds are likely to reside in the ER as a result of the suppression of retrograde transport of these proteins to the cytoplasm. Thus, the unfolded protein response (UPR) was evaluated in *OsHrd3* KD seeds. Mature seeds from *OsHrd3* KD plants displayed an abnormal phenotype, with a slightly floury and shrunken appearance (Supplementary Fig. S2A at *JXB* online), and the grain weight of the *OsHrd3* KD seeds was significantly lower than that of WT seeds (*P*=0.002 by *t*-test, Supplementary Fig. S2B), suggesting that the UPR occurred in these seeds.

To ascertain that the UPR was induced in the *OsHrd3* KD seeds, the levels of ER-resident chaperones in mature seeds were investigated. Immunoblot analyses demonstrated that the levels of UPR-responsive OsBiP4 and OsBiP5 (Wakasa *et al.*, 2012) were higher in the *OsHrd3* KD seeds than in the WT (Fig. 3A). Furthermore, the levels of OsBiP1 and OsPDIL2-3 were also higher in the *OsHrd3* KD seeds than in the WT (Fig. 3A). See Supplementary Fig. S2C at *JXB* online for a loading control of this experiment. RT-PCR analysis revealed unconventional splicing of *OsbZIP50* mRNA (Hayashi *et al.*, 2012) in *OsHrd3* KD seeds at 21 DAF concomitant with the induction of *OsBiP4* (Fig. 3B). These results indicate that the OsIRE1/OsbZIP50 signalling pathway for the UPR was activated in *OsHrd3* KD seeds and that the OsIRE1/OsbZIP50 signalling pathway partly accounts for the ER stress responses in *OsHrd3* KD seeds. In contrast, the transcript levels of *OsbZIP39* and *OsbZIP60* were not affected in *OsHrd3* KD seeds (Fig. 3B). Thus, the UPR is induced in *OsHrd3* KD seeds, implying that the unfolded proteins reside in the ER of *OsHrd3* KD seeds and that *OsHrd3* is necessary for removing unfolded proteins from the ER in rice endosperm.

### Accumulation of aberrant aggregations in *OsHrd3* KD seeds

**Fig. 4.**
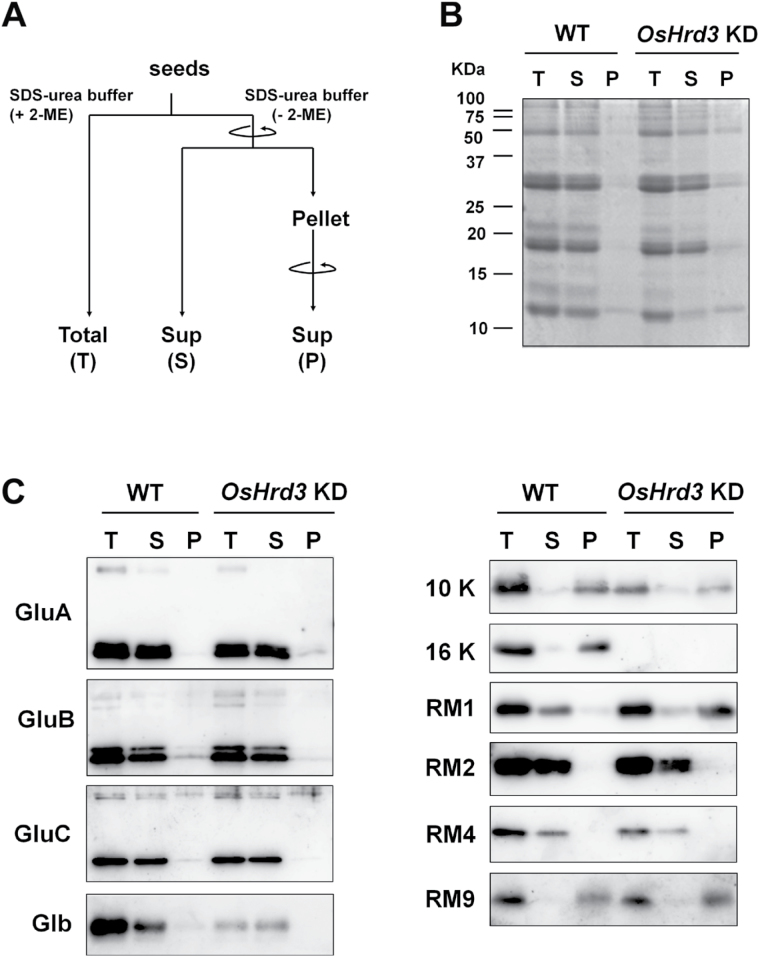
Aberrant aggregation of RM1 in *OsHrd3* KD seeds. (A) Schematic representation of the experiment. Total proteins were extracted from mature wild-type (WT) and *OsHrd3* KD seeds with SDS–urea buffer without 2-mercaptoethanol and fractionated into the supernatant (S) and pellet (P) by centrifugation. The resulting pellets were again extracted with SDS–urea buffer containing 2-mercaptoethanol to collect the solubilized proteins, and proteins in the S fraction were denatured in the presence of 2-mercaptoethanol. For a control, total proteins (T) were extracted from mature WT and *OsHrd3* KD seeds with SDS–urea buffer supplemented with 2-mercaptoethanol. (B) SDS–PAGE analysis of the T, S, and P fractions derived from WT and *OsHrd3* KD seeds. Total proteins of the T, S, and P fractions were subjected to immunoblot analyses using antibodies against rice seed storage proteins. (C) Immunoblot analysis of the total (T), soluble (S), and pellet (P) fractions derived from WT and *OsHrd3* KD seeds. Total proteins of the T, S, and P fractions were subjected to immunoblot analyses using antibodies against rice seed storage proteins.

To compare the levels of SSPs in *OsHrd3* KD versus WT mature seeds, proteins were extracted from these seeds using SDS–urea buffer containing 2-mercaptoethanol (fraction T in Fig. 4A). Immunoblot analysis showed that the accumulation of 26kDa globulin (Glb) and 16kDa prolamin (16 k) was dramatically reduced in the *OsHrd3* KD seeds (Fig. 4C). The mRNA levels for *Glb* and *16 k* were also down-regulated in maturing seeds (14–21 DAF) from *OsHrd3* KD (Supplementary Fig. S2D at *JXB* online), implying that the reduced accumulation of Glb and 16 k was related to a decrease in transcript levels. Although levels of 10kDa prolamin (10 k) and 13kDa prolamin, RM4, were also decreased in the *OsHrd3* KD seeds, the levels of other SSPs examined here were not affected in the *OsHrd3* KD seeds (Fig. 4C).

Unfolded proteins in mammalian cells tend to form aggregations through cross-linking of interchain disulphides (Machamer and Rose, 1988). To assess this possibility in plants, proteins were extracted from mature seeds using SDS–urea buffer without 2-mercaptoethanol to maintain the aggregation of unfolded proteins caused by aberrant disulphide bond formation (Fig. 4). Most of the SSPs from both WT and *OsHrd3* KD seeds were detected in the soluble (S) fraction (Fig. 4). In contrast, cysteine-rich proteins such as 10kDa prolamin (10 k), 13kDa prolamin (RM9), and 16kDa prolamins (16 k) were detected in the pellet (P) fractions from both WT and *OsHrd3* KD seeds (Fig. 4C). Although another cysteine-rich 13kDa prolamin, RM1, was detected in the S fraction from WT seeds, most of the RM1 in *OsHrd3* KD seeds was detected in the P fraction (Fig. 4C). This result suggests that the cysteine residues of RM1 form aberrant disulphide bonds in *OsHrd3* KD seeds. These results imply that OsHrd3 is required for the removal of unfolded SSPs, such as RM1.

### Deformed PB-I in *OsHrd3* KD seeds

**Fig. 5.**
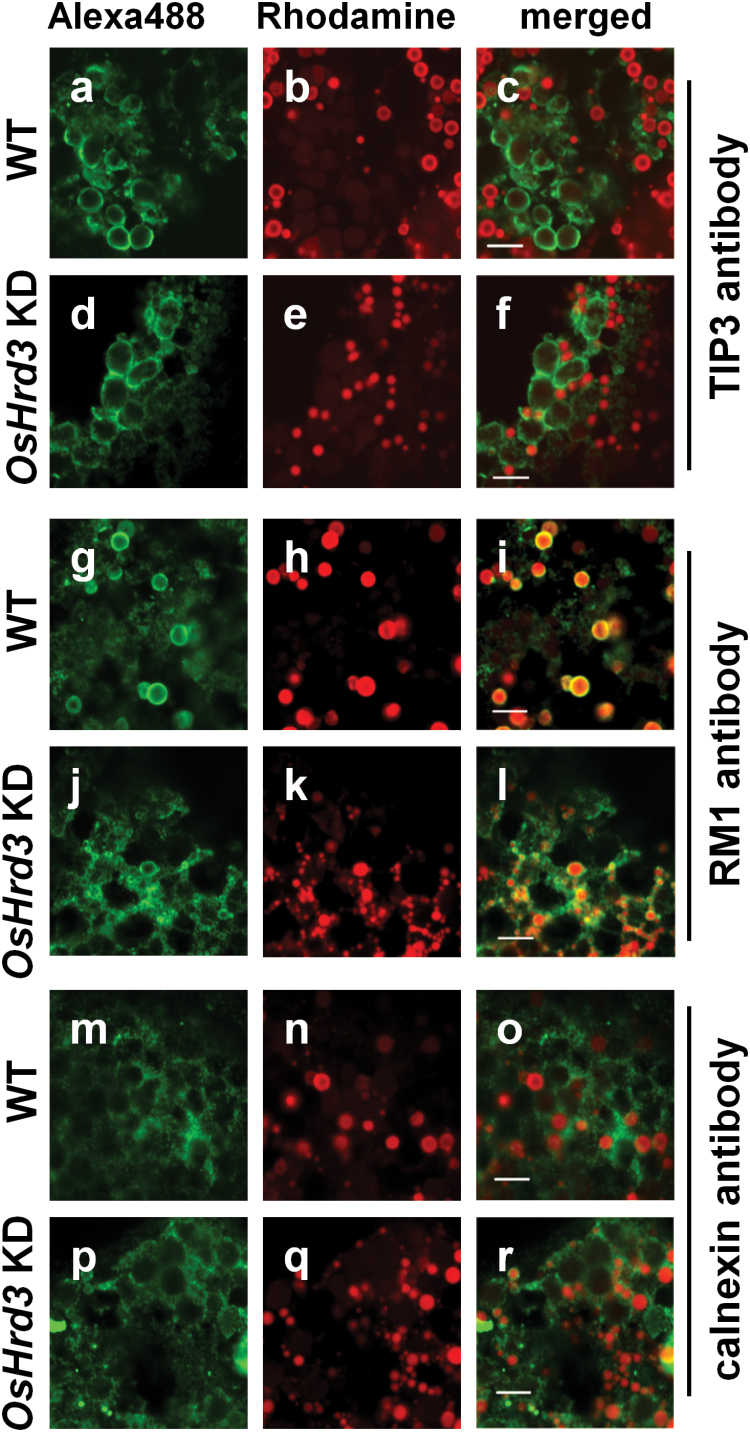
Aberrant protein body-I (PB-I) in the *OsHrd3* KD seeds. Indirect immunohistochemical analysis of rice endosperm (18 DAF) using antibodies against OsTIP3 (a–f), RM1 (g–l), and calnexin (m–r). Signals were detected using a secondary antibody conjugated with Alexa488, which emits green fluorescence (a, d, g, j, m, p). PB-I was stained with rhodamine B (b, e, h, k, n, q). Right panels (c, f, i, l, o, r) are merged images of the left (green fluorescence) and the middle (red fluorescence) panels. Scale bar=5 μm.

To investigate whether the intracellular structure and protein body formation were altered in *OsHrd3* KD seeds, indirect immunohistochemical analysis was carried out using confocal microscopy (Fig. 5). The staining pattern of the PB-II marker TIP3 antibody (Takahashi *et al.*, 2004) in *OsHrd3* KD seeds was almost the same as that in WT seeds (Fig. 5a, c, d, f). In contrast, rhodamine staining revealed that PB-I was smaller in *OsHrd3* KD seeds than in WT seeds (Fig. 5b, c, e, f). These results demonstrate that PB-I, but not PB-II, is severely affected in *OsHrd3* KD seeds.

RM1 was detected in the periphery of PB-I in WT seeds (Fig. 5g, i). In contrast, in *OsHrd3* KD seeds, RM1 was detected not only around the periphery of PB-I but also on mesh-like structures that were connected to PB-I (Fig. 5j, l). These mesh-like structures were likely to be ER lumen because the staining pattern of an ER marker, obtained using an antibody against calnexin (CNX; Fig. 5m, o, p, r), was very similar to the RM1 staining pattern observed in *OsHrd3* KD seeds. It should be noted that CNX was seldom detected around PB-I in WT seeds (Fig. 5m, o), whereas CNX was observed around PB-I in *OsHrd3* KD seeds (Fig. 5p, r). These results demonstrate the aberrant distribution of RM1 and the deformation of PB-I in *OsHrd3* KD seeds.

### OsHrd3 is required for ubiquitination of RM1

**Fig. 6.**
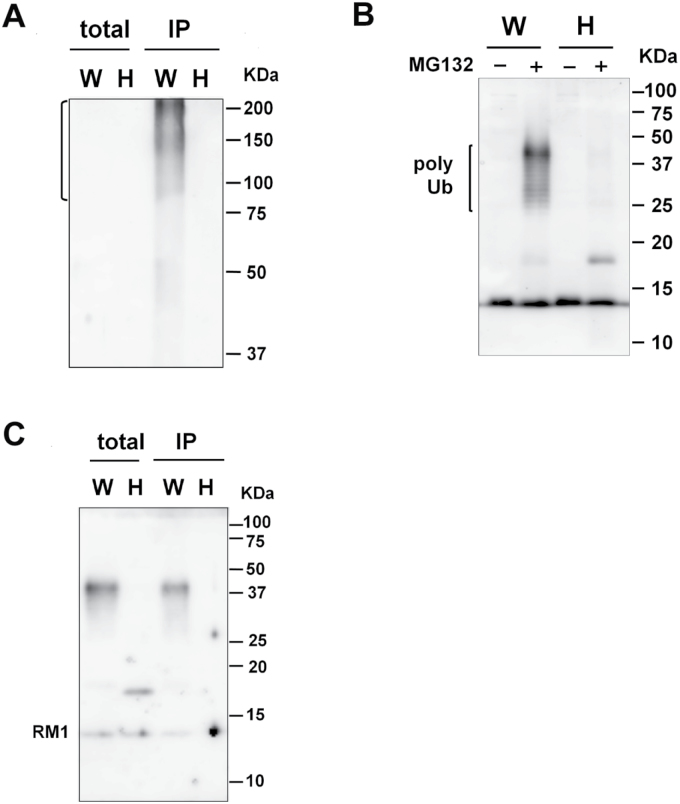
OsHrd3 is required for polyubiquitination of RM1. Seeds (14 DAF) from wild-type (WT) and *OsHrd3* KD plants were dehulled and treated with 100 μM MG132 for 24h, and then with 20 μM PR-619 for 1h. (A) Total proteins were extracted with SDS–urea buffer supplemented with 2-mercaptoethanol and separated by SDS–PAGE, followed by immunoblot analyses using antibodies against RM1. Total proteins were extracted from the seeds and then immunoprecipitated with antibody against ubiquitin–protein conjugates. Total proteins (2%) and immunoprecipitated proteins (IP) were separated by SDS–PAGE, followed by immunoblot analyses using antibodies against the ubiquitin–protein conjugates (B) and RM1 (C). W and H represent samples from WT and *OsHrd3* KD seeds, respectively.

The results reveal that RM1 formed aberrant aggregations in *OsHrd3* KD seeds (Fig. 4). However, other cysteine-rich prolamins [such as 10kDa prolamin, 13kDa prolamin (RM9), and 16kDa prolamin] did not form such aberrant aggregations in *OsHrd3* KD seeds (Fig. 4). Since the polyubiquitination of unfolded proteins was reduced in *OsHrd3* KD seeds (Figs 2B, 4), RM1 is the most plausible candidate for a protein that is mislocated into the cytoplasm as an unfolded/misfolded protein and ubiquitinated by the Hrd1 ubiquitin ligase complex containing OsHrd3. To explore this possibility, the polyubiquitination of RM1 was examined (Fig. 6). When the dehulled seeds (14 DAF) were treated with MG132 and then with a deubiquitinase inhibitor, PR-619 (Seiberlich *et al.*, 2012), RM1 with a higher molecular weight (ranging from 30kDa to 50kDa) was detected in MG132-treated samples from WT seeds but not from *OsHrd3* KD seeds (Fig. 6A). To confirm whether the higher molecular weight RM1 was ubiquitinated, an immunoprecipitation experiment was carried out using anti-ubiquitinated proteins. RM1 is usually deposited in insoluble PB-I. To carry out immunoprecipitation experiments, it was important first to examine whether RM1 could be solubilized in extraction buffer. Some RM1 was detected in the soluble fraction (S3) from the WT, developing seeds (Supplementary Fig. S3A at *JXB* online). Furthermore, RM1 was detected in the microsomal fraction, which could be solubilized by 1% Triton X-100 (Supplementary Fig. S3B). Therefore, seed proteins were extracted with buffer containing Triton X-100. Total proteins were extracted from the MG132-treated seeds with a buffer containing 0.5% Triton X-100. Consistent with Fig. 2B, the levels of polyubiquitinated proteins in WT seeds were higher than those in *OsHrd3* KD seeds (Fig. 6B). RM1 was detected in total extracts before immunoprecipitation, indicating that RM1 from both WT and *OsHrd3* KD seeds was extracted with buffer containing 0.5% Triton X-100. As shown in Fig. 6C, the higher molecular weight RM1 was immunoprecipitated by the anti-ubiquitinaed protein only from the WT seeds but not from *OsHrd3* KD seeds (Fig. 6C). Mono RM1 that had not been modified by ubiquitin was also found only in WT immunoprecipitated samples. Since mature RM1 does not have lysine residues, which are authentic acceptor sites of ubiquitin, other residues such as cysteine and serine are likely to serve as ubiquitin acceptor sites. The mono RM1 observed in the WT may have been derived from polyubiquitinated RM1 by partial cleavage of the disulphide bond between cysteine and ubiquitin during denaturation under reducing conditions. Thus, these results demonstrate that OsHrd3 is involved in the ubiquitination of RM1 in rice endosperm.

## Discussion

Hrd3/EBS5 was first identified as a suppressor of the *Arabidopsis* brassinosteroid-insensitive mutant *bri1-9* (Su *et al.*, 2011). Salt stress induces the UPR in *Arabidopsis*, and *ebs5-1/hd3a-1* also exhibits increased salt sensitivity (Liu *et al.*, 2011). Interestingly, the levels of polyubiquitinated proteins are higher in *ebs5-1/hd3a-1* than in WT plants, raising the possibility that unfolded proteins produced during salt stress are ubiquitinated by other ERAD ubiquitin ligases such as *Arabidopsis* homologues of Doa1/TEB4 and gp78 (Liu *et al.*, 2011). The current study identified an SSP, RM1, as another substrate of Hrd3 in plants. In contrast to *Arabidopsis* plants, the level of polyubiquitinated proteins was dramatically reduced in rice *OsHrd3* KD seeds (Figs 2B, 6A). Thus, the Hrd1 ubiquitin ligase complex is likely to be a major ERAD ubiquitin ligase in rice endosperm.

Unfolded proteins are produced in seeds in several different ways, and they affect the formation and accumulation of SSPs. For example, the expression of recombinant proteins such as human β-amyloid and human interleukin 7 (hIL-7) induces the UPR in transgenic rice seeds, and accumulation of SSPs is reduced in these transgenic seeds (Oono *et al.*, 2010; Kudo *et al.*, 2013). Moreover, genetic mutations in zeins, the major SSPs of maize, stimulate UPRs in maize endosperm (Coleman *et al.*, 1997; Kim *et al.*, 2004, 2006). These studies reveal the importance of a protein quality control system to remove proteins that are unfolded due to the fact that they are foreign or mutant proteins. In contrast, as demonstrated in the current study, WT proteins can become unfolded even under normal conditions, since the UPR was induced in *OsHrd3* KD seeds and unfolded proteins formed aggregations in these seeds. The results reveal that protein quality control is important for maintaining healthy conditions in rice endosperm even under normal conditions.

It was found that the expression of *OsHrd3* is essential for polyubiquitination of RM1. This result suggests that unfolded RM1 is mislocated into the cytosol and ubiquitinated by Hrd1 ubiquitin ligase due to the loss of OsHrd3 function. The exact mechanism of how OsHrd3 affects the ubiquitin ligase activity of OsHrd1 is unknown. One possibility is that Hrd3 affects the stability of Hrd1, as shown in yeast (Plemper *et al.*, 1999; Gardner *et al.*, 2000). Consistent with the yeast Hrd1p complex, Hrd1-HA was detected in rice protoplasts only when Hrd3-FLAG was co-transfected with Hrd1-HA (Supplementary Fig. S4 at *JXB* online). Rice Hrd1 ubiquitin ligase may be destabilized in *OsHrd3* KD seeds and, consequently, the level of polyubiquitinated proteins is reduced in these seeds (Figs 2B, 6A). Another possibility is that substrate recognition is impaired in *OsHrd3* KD seeds, as Hrd3/SEL1L is involved in substrate recognition in yeast and mammalian cells (Lilley and Ploegh, 2005; Carvalho *et al.*, 2006; Denic *et al.*, 2006).

The formation of multiprotein complexes potentially leads to aggregation because subunits are likely to have exposed hydrophobic aggregation-prone surfaces and long unstructured regions, which mediate protein–protein interactions among the subunits (Tsai *et al.*, 2009). If the level of one subunit exceeds that of the other subunits, the excess unassembled subunit may bind to other proteins, thereby interfering with their functions. Therefore, the excess subunits must be quickly removed by a cellular protein control system. For example, the ζ subunits of the T-cell receptor complex are a limiting factor for assembly, and >70% of the other subunits remain unassembled and degraded without reaching the cell surface (Minami *et al.*, 1987; Sussman *et al.*, 1988). Thus, stoichiometric co-ordination of subunit abundance and protein quality control of excess subunits are essential for the proper formation of multiple subunit complexes. RM1 is localized in the middle layer of PB-I between the central core cysteine-rich 10kDa prolamin and the peripheral cysteine-depleted 13kDa prolamin (Saito *et al.*, 2012), suggesting that RM1 can interact with multiple proteins and form aggregations through disulphide bonds between these proteins. Unfolded proteins are cross-linked by interchain disulphides in mammalian cells (Machamer and Rose, 1988). Indeed, *OsHrd3* KD seeds accumulated aberrant aggregations of RM1 and induced the UPR (Figs 2, 3,d 5). Thus, it is proposed that the Hrd1 ERAD system is required for removing unassembled RM1 from the ER, and that protein quality control by the Hrd1 ERAD system provides a mechanism for co-ordinating the abundance of prolamins in PB-I. It has been proposed that the concentric layered structure of rice prolamins is formed by temporal regulation of prolamin genes during seed development (Saito *et al.*, 2012). The present proposal provides insights into the importance of protein quality control in the formation of PB-I in rice endosperm.

In addition to RM1, rice cysteine-rich prolamins also include 10kDa prolamin, 13kDa prolamin (RM9), and 16kDa prolamin. The 10kDa prolamin initially accumulates and forms a core inside PB-I, and other cysteine-rich prolamins are subsequently synthesized to cover the core 10kDa prolamin (Saito *et al.*, 2012). This differential expression of 10kDa and other prolamins enables free cysteine residues of 10kDa prolamin to be masked by other cysteine-rich prolamins through proper disulphide bond formation. Consequently, the risk of formation of aberrant S–S bonds between the 10kDa prolamin and other non-relevant proteins may be reduced. Thus, it is speculated that 10kDa prolamin levels are not necessarily regulated by the Hrd1 ubiquitin ligase complex. In contrast to 10kDa prolamin, RM9 and 16kDa prolamin may have similar properties to RM1 and may therefore be ubiquitinated by the Hrd1 ubiquitin ligase complex if these prolamins overaccumulate in rice endosperm. However, the possibility cannot be ruled out that RM9 and 16kDa prolamin are ubiquitinated by other ubiquitin ligases. This possibility was examined; however, it was difficult to analyse the ubiquitination of RM9 and 16kDa prolamin because RM9 or 16kDa prolamin could not be solubilizes, even with SDS (Fig. 4). Developing methods to analyse the ubiquitination of insoluble proteins may help shed light on the protein quality control system in rice endosperm.

## Supplementary data

Supplementary data are available at *JXB* online.

Figure S1. Subcellular localization and membrane topology of OsHrd3 in rice protoplasts.

Figure S2. *OsHrd3* KD seeds show unfolded protein responses.

Figure S3. Fractionation of RM1 from maturing seeds.

Figure S4. OsHrd3 is necessary for the accumulation of OsHrd1.

Table S1. A list of primers used in this study

Supplementary Data

## Abbreviations:

BiP: immunoglobulin binding protein
Co-IP: co-immunoprecipitation
ER: endoplasmic reticulum
ERAD: ER-associated degradation
Hsp70: heat shock protein
IL: interleukin
PB: protein body.
