# Supplementary material for: OsHrd3 is necessary for maintaining the quality of endoplasmic reticulum-derived protein bodies in rice endosperm
Source: J Exp Bot. 2015 May 14;66(15):4585–93. doi: 10.1093/jxb/erv229 (PMC4507767; doi:10.1093/jxb/erv229)
Supplement: Supplementary Data [file supp_66_15_4585__index.html]

OsHrd3 is necessary for maintaining the quality of endoplasmic reticulum-derived protein bodies in rice endosperm — OsHrd3 is necessary for maintaining the quality of endoplasmic reticulum-derived protein bodies in rice endosperm — Supplementary Data 

# OsHrd3 is necessary for maintaining the quality of endoplasmic reticulum-derived protein bodies in rice endosperm

## Supplementary Data

Data files

**Files in this Data Supplement:**

- Supplementary Data - Supplementary Data
