# Supplementary material for: OsHrd3 is necessary for maintaining the quality of endoplasmic reticulum-derived protein bodies in rice endosperm
Source: J Exp Bot. 2015 May 14;66(15):4585–93. doi: 10.1093/jxb/erv229 (PMC4507767; doi:10.1093/jxb/erv229)
Supplement: Supplementary Data [file supp_erv229_jexbot145243_file001.pdf]

Supplemental data

OsHrd3 is necessary for maintaining the quality of ER-derived protein bodies in rice endosperm

Masaru Ohta and Fumio Takaiwa*

***Corresponding author:** Fumio Takaiwa

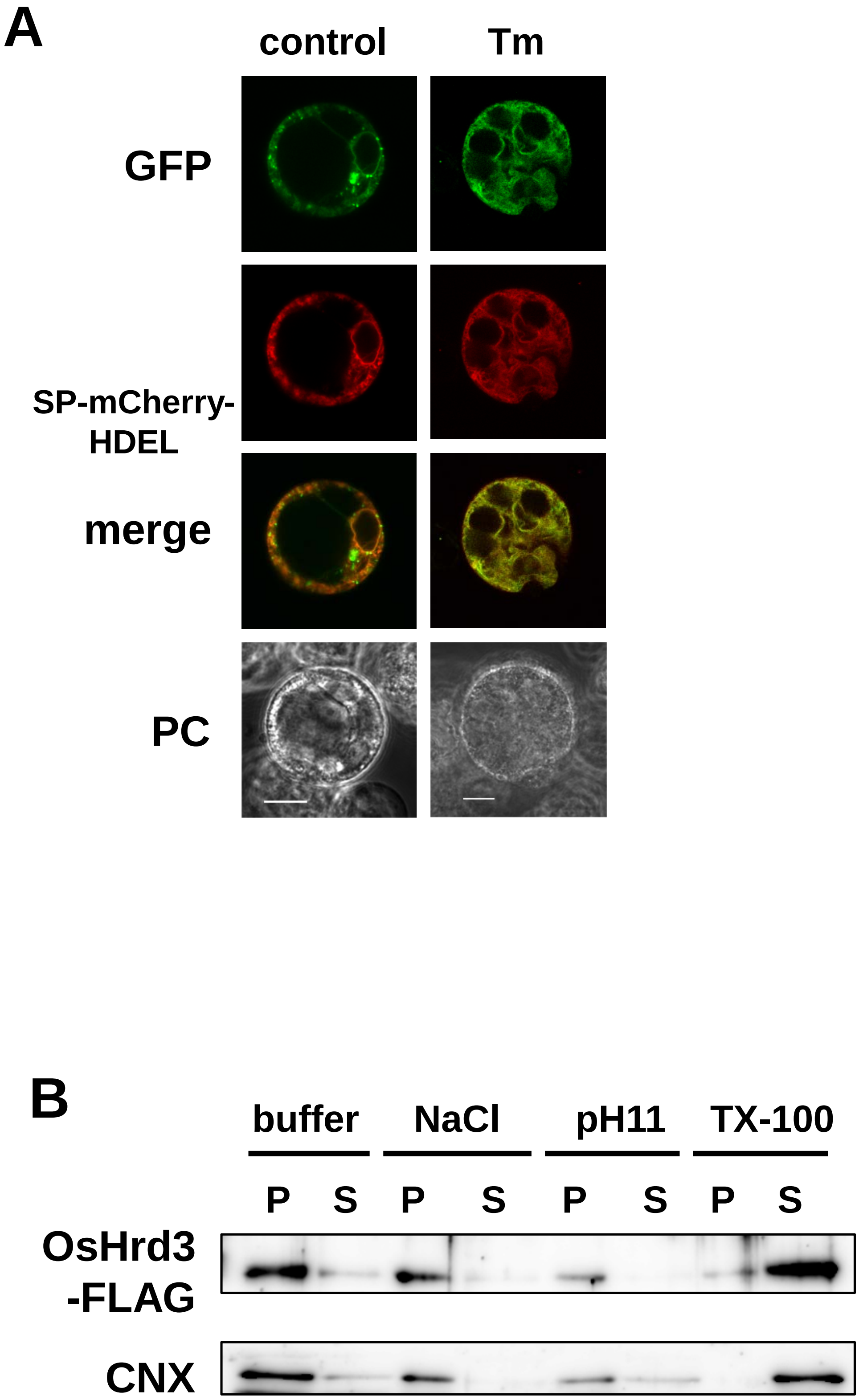
A
control
Tm
GFP
SP-mCherry-
HDEL
merge
PC
B
buffer
NaCl
pH11
TX-100
P S P S P S P S
OsHrd3
-FLAG
CNX

Fig. S1.

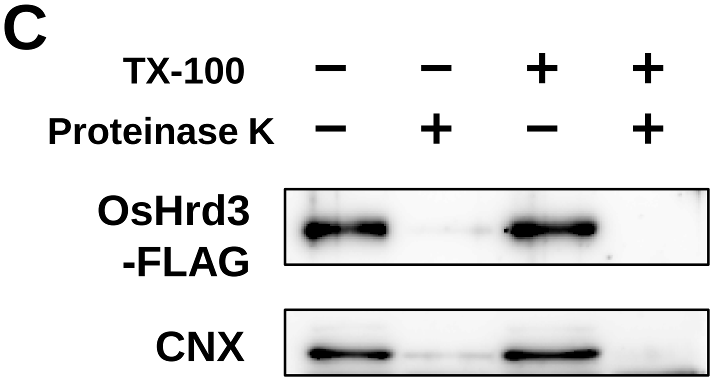

Fig. S1. Sub-cellular localization and membrane topology of OsHrd3 in rice protoplasts.
(A) OsHrd3-GFP fusion protein co-localizes with an ER-marker protein in protoplasts. An ER-localized mCherry (SP-mCherry-HDEL) fusion construct was transiently co-transfected with OsHrd3-GFP in rice protoplasts prepared from rice suspension culture cells (Oc cells). After 18 h of incubation, the protoplasts were treated with 0.1% DMSO (control) or 5 μg mL-1 tunicamycin (Tm) for an additional 6 h. The cells were observed and photographed with a confocal laser-scanning microscope. Bar = 10 □m. (B) Analysis of microsomal fraction from rice protoplasts expressing *OsHrd3-FLAG* tag fusion proteins. The microsomal fraction was suspended in control buffer, high salt buffer (1M NaCl), alkaline buffer (100 mM Na2CO3, pH 11), and Triton X-100 buffer (1% Triton X-100). These suspensions were ultracentrifuged to obtain the pellet (P) and supernatant (S) fractions. Each fraction was subjected to immunoblot analysis with anti-FLAG-HRP conjugated or anti-calnexin (CNX) antibodies. (C) Protease treatment of the microsomal fraction from rice protoplasts expressing *OsHrd3-FLAG.*

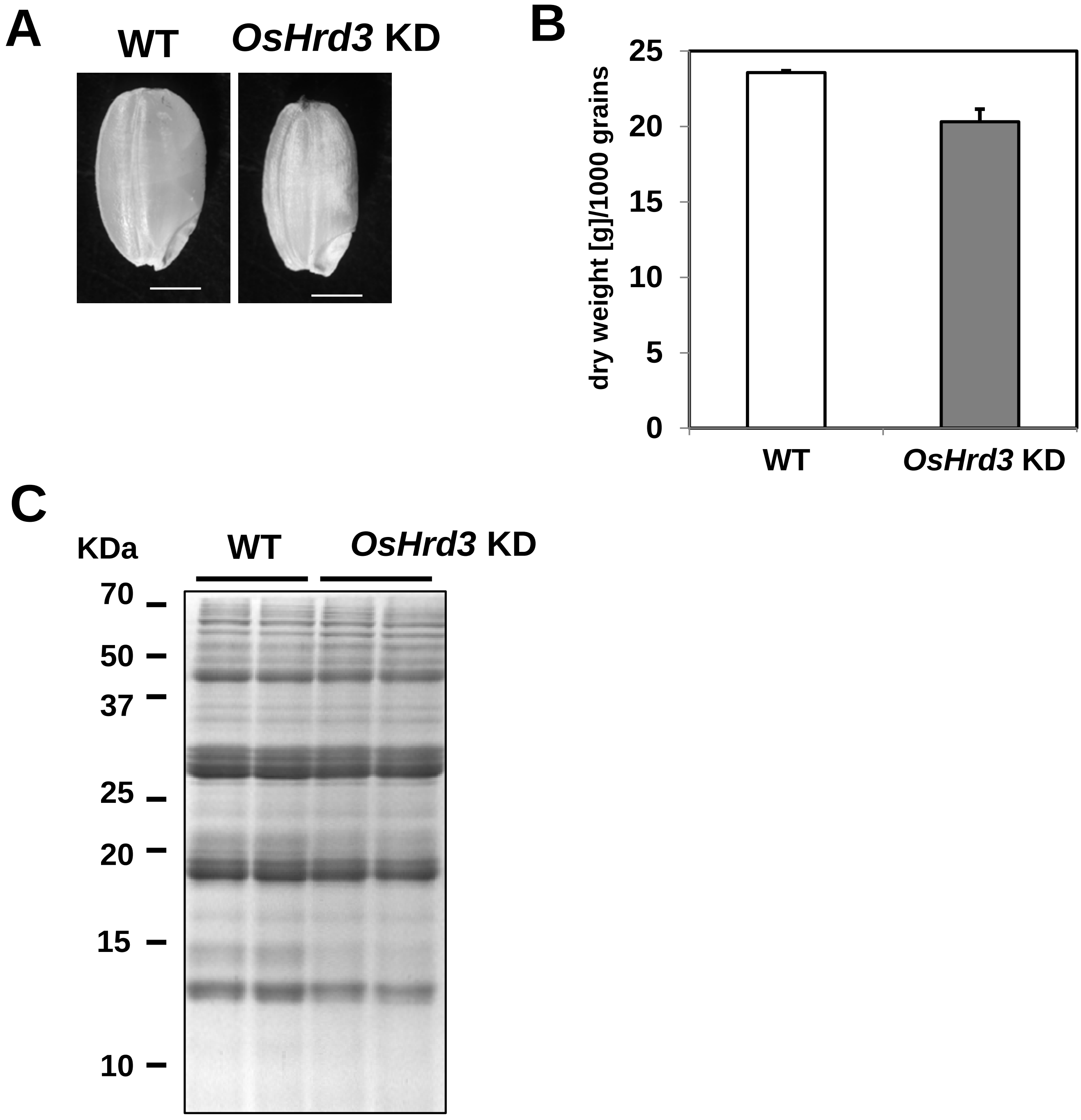
A
WT
OsHrd3 KD
B
25
20
15
10
5
0
dry weight [g]/1000 grains
WT
OsHrd3 KD
C
KDa
WT
OsHrd3 KD
70
50
37
25
20
15
10

Fig. S2.

D

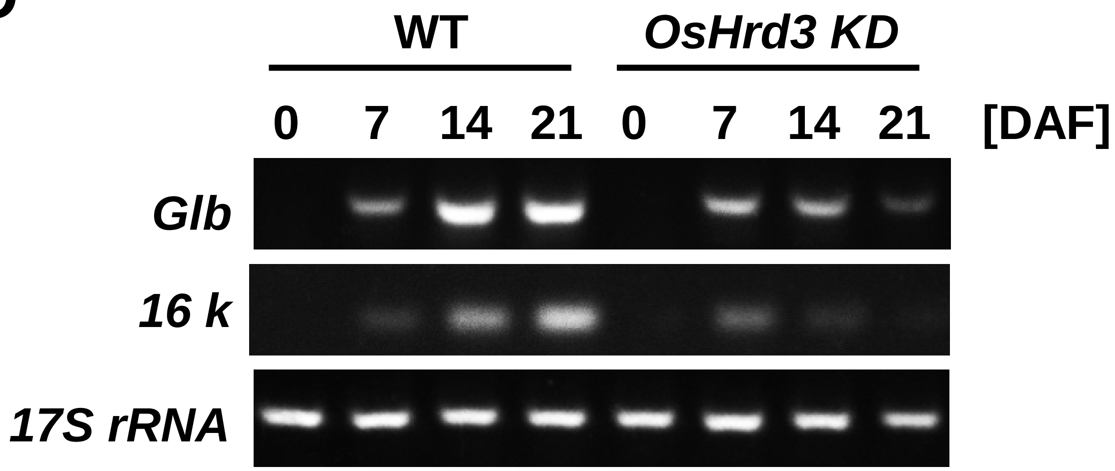

Fig. S2. OsHrd3 KD seeds show unfolded protein responses.
(A) Phenotypes of wild-type (WT) and OsHrd3 KD seeds. Bar = 2 mm. (B) Dry weight (g/1000 seeds) of mature wild-type and OsHrd3 KD seeds. Error bars represent SD from three replicates harvested different years. (C) SDS-PAGE analysis of total proteins extracted from mature WT and OsHrd3 KD seeds. Total proteins were extracted from mature seeds with SDS-urea buffer containing 2-ME. The total proteins were separated by SDS-PAGE, followed by immunoblot analyses using antibodies against ER-resident chaperones. (D) RT-PCR analysis of the transcript levels of genes encoding 26 kDa globulin (Glb) and Cys-rich 16 kDa prolamin (16 k). Total RNAs were isolated from seed tissues at 0, 7, 14 and 21 DAF. 17S rRNA was analyzed as a loading control.

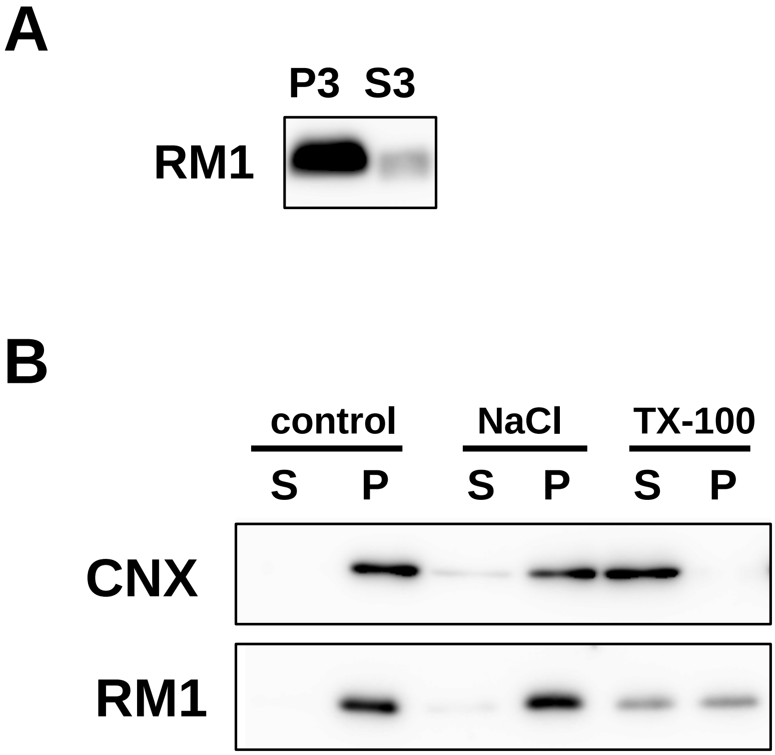

Fig. S3. Fractionation of RM1 from maturing seeds. (A) Some RM1 was present in the S3 fraction. Total homogenates from wild-type 14 DAF seeds were centrifuged at 3,000 g to fractionate them into the supernatant (S3) and pellet (P3). (B) The S3 fraction was subsequently centrifuged at 100,000 g under the following conditions: control buffer, a buffer containing 1 M NaCl buffer, or 1% Triton X-100 (TX-100). The supernatant (S) and pellet (P) fractions were separated by SDS-PAGE, followed by immunoblot analyses using antibodies against CNX and RM1.

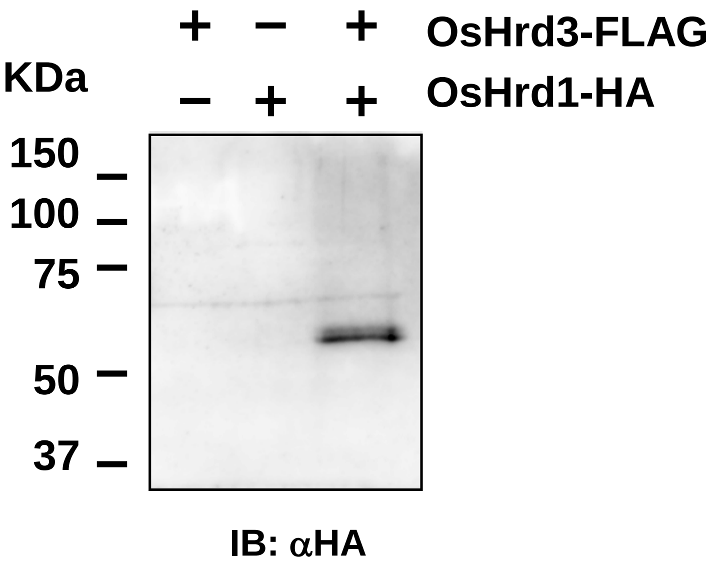

Fig. S4. OsHrd3 is necessary for the accumulation of OsHrd1. Protoplasts expressing OsHrd1-HA and/or OsHrd3-FLAG were extracted with SDS loading buffer. The total proteins were separated by SDS-PAGE, followed by immunoblot analyses using anti-HA-HRP conjugated antibody.

Table S1. Primers used for plasmid construction and RT-PCR analysis

Plasmid construction

Construct name	primer	sequence
OsHrd3-GFP	Hrd3-9	5'-TAACCCGGGATGCAGAACAGGCGGCGATCTC-3'
	Hrd3-10	5'-GTTGCCCGGGGCATGGGTGCACCATCAGGCTG-3'
Ubip-2xHA-GluBter	HA-3	5'-GCGGGTACCTACCCATACGACGTTCCAGACTACGCTGGTTACCCATACGAC-3'
	HA-4	5'-CTGGAGCTCAACCGGATCTAGCGTAGTCTGGAACGTCGTATGGGTAACC-3'
OsHrd1-HA	Hrd1-3	5'-GAATGGTACCATGATTCGGCTGCAGACGTACG-3'
	Hrd1-4	5'-GAATTGGTACCGTCGCTTTTTGTGTGCTCACC-3'
OsOS9-HA	OS9-3	5'-GTCGCCGACCATGGGGCTCGCCG-3'
	OS9-4	5'-AATGATGCATACCATGGTAGCATAAGCTG-3'
OsEDEM1-HA	EDEM1-1	5'-GAGCATGCCCACCTCGGGCCCGGAC-3'
	EDEM1-2	5'-CCTTGGATCCGGTCAGGTATCTTCCTTCGGTCG-3'
OsHrd3-RNAi	Hrd3-1	5'-TGCTCTAGAGCTTGCACTAACAAGTTTATGG-3'
	Hrd3-2	5'-TGCTCTAGAAGTATACGTTTTCTTGAACAAC-3'
	Hrd3-3	5'-AGAGGATCCGCTTGCACTAACAAGTTTATGG-3'
	Hrd3-4	5'-AGAGGATCCAGTATACGTTTTCTTGAACAAC-3'

RT-PCR

Gene name	primer	sequence
OsHrd3	Hrd3-7	5'-TTCTCAGCCATATCATACCATC-3'
	Hrd3-8	5'-GGAGAGCTATCATTTTAAACCC-3'
Glb	Glb-1	5'-TCGTCGGAGGAGGGCTACTAC-3'
	Glb-2	5'-ATCTCGTTGCATGCAACACAG-3'
16 k	16K-3	5'-ATGGCGCAACAATATCATTGC-3'
	16K-2	5'-ATGGTACACACTACCAAGAAC-3'
